# Trypanosome Infection Establishment in the Tsetse Fly Gut Is Influenced by Microbiome-Regulated Host Immune Barriers

**DOI:** 10.1371/journal.ppat.1003318

**Published:** 2013-04-18

**Authors:** Brian L. Weiss, Jingwen Wang, Michele A. Maltz, Yineng Wu, Serap Aksoy

**Affiliations:** Department of Epidemiology of Microbial Diseases, Yale School of Public Health, New Haven, Connecticut, United States of America; University of Wisconsin-Madison, United States of America

## Abstract

Tsetse flies (*Glossina* spp.) vector pathogenic African trypanosomes, which cause sleeping sickness in humans and nagana in domesticated animals. Additionally, tsetse harbors 3 maternally transmitted endosymbiotic bacteria that modulate their host's physiology. Tsetse is highly resistant to infection with trypanosomes, and this phenotype depends on multiple physiological factors at the time of challenge. These factors include host age, density of maternally-derived trypanolytic effector molecules present in the gut, and symbiont status during development. In this study, we investigated the molecular mechanisms that result in tsetse's resistance to trypanosomes. We found that following parasite challenge, young susceptible tsetse present a highly attenuated immune response. In contrast, mature refractory flies express higher levels of genes associated with humoral (*attacin* and *pgrp-lb*) and epithelial (*inducible nitric oxide synthase* and *dual oxidase*) immunity. Additionally, we discovered that tsetse must harbor its endogenous microbiome during intrauterine larval development in order to present a parasite refractory phenotype during adulthood. Interestingly, mature aposymbiotic flies (*Gmm* ^Apo^) present a strong immune response earlier in the infection process than do WT flies that harbor symbiotic bacteria throughout their entire lifecycle. However, this early response fails to confer significant resistance to trypanosomes. *Gmm* ^Apo^ adults present a structurally compromised peritrophic matrix (PM), which lines the fly midgut and serves as a physical barrier that separates luminal contents from immune responsive epithelial cells. We propose that the early immune response we observe in *Gmm* ^Apo^ flies following parasite challenge results from the premature exposure of gut epithelia to parasite-derived immunogens in the absence of a robust PM. Thus, tsetse's PM appears to regulate the timing of host immune induction following parasite challenge. Our results document a novel finding, which is the existence of a positive correlation between tsetse's larval microbiome and the integrity of the emerging adult PM gut immune barrier.

## Author Summary

Tsetse flies serve as a host to many micro-organisms. Specifically, this fly houses beneficial endosymbiotic bacteria, and can also serve as a vector of pathogenic trypanosomes across much of sub-Saharan Africa. Although flies feed on parasite-infected reservoir hosts, only a small proportion (1–5%) of individuals that acquire an infectious meal become infected and subsequently transmit disease to a naïve host. Several physiological factors, including tsetse's age, nutritional status and innate immune mechanisms, contribute to trypanosome infection outcomes in the fly. We demonstrate that tsetse's endogenous microbiome also impacts the fly's resistance to parasites. Specifically, we show that tsetse must harbor it's symbiotic bacteria during larval development in order to present a trypanosome-refractory phenotype during adulthood. These microbes appear to indirectly regulate the fly's ability to immunologically detect and respond to the presence of trypanosomes. One of the mechanisms by which these microbes regulate parasite transmission involves modulating the formation of a physical barrier (called the ‘peritrophic matrix’) in their host's gut. Our findings are indicative of the complex functional association that exists between tsetse's symbiotic microbes and host immune mechanisms that regulate trypanosome infection outcomes.

## Introduction

Tsetse flies (*Glossina* spp.) serve as the sole vector of protozoan African trypanosomes (*Trypanosoma brucei* spp.), which are the causative agents of Human African trypanosomiasis (HAT), or sleeping sickness, throughout most of sub-Saharan Africa. Additionally, parasites from this same species complex also infect domesticated animals, causing an economically devastating disease called nagana. During their lifecycle through mammalian and tsetse hosts, African trypanosomes undergo a genetically complex differentiation process. Once in the fly, stumpy form mammalian trypanosomes differentiate to become procyclics [1], [2]. At this point most tsetse hosts can efficiently clear their infections [3]. In fact, despite the large number of infected animal reservoirs and high disease burden in Africa, relatively few tsetse flies (<5%) are able to successfully transmit trypanosomes to susceptible mammalian hosts [4]. Furthermore, even under ideal laboratory-based conditions, only a small proportion of adult flies are able to transmit parasites to a naïve host [4], [5].

Several physiological factors have been identified that may contribute to tsetse's natural trypanosome refractory phenotype. These include fly age and nutritional status at the time of exposure to infectious trypanosomes [6]–[8], antimicrobial peptides (AMPs) [9], [10], trypanosome-binding lectins [11], [12], gut-associated EP protein [13], [14], reactive oxygen species (ROS) [15], [16] and parasite inhibitory peptidoglycan recognition protein LB (PGRP-LB) [17], [18].

Many insects that transmit mammalian disease also house gut-associated microbes that modulate their vector competence [19]–[21]. In anopheline mosquitoes, malaria infection outcomes can be directly modulated by the host gut microbiome. For example, commensal bacteria (*Enterobacter* spp.) found naturally in the *Anopheles gambiae* midgut produce reactive oxygen species that directly inhibit *Plasmodium* development [22]. Alternatively, commensal fauna in the mosquito gut can indirectly regulate infection outcomes by boosting host immunity, which in turn detrimentally impacts pathogen transmission. This phenomenon was observed when malaria infection outcomes were observed in septic and aseptic adult *A. gambiae* flowing challenge with *Plasmodium* gametocytes. Specifically, adult mosquitoes that lacked their microbiome displayed an increased susceptibility to parasites, while their counterparts that housed endogenous bacteria were highly resistant [23], [24]. These high infection outcomes were attributed to the absence of microbiome-induced anti-*Plasmodium* immune responses in aseptic mosquitoes. Tsetse flies harbor 3 distinct endosymbiotic bacteria that are intimately associated with their host's physiology. These symbionts, obligate *Wigglesworthia*, commensal *Sodalis* and parasitic *Wolbachia*, are maternally transmitted during tsetse's unique viviparous mode of reproduction [25], [26]. Unlike mosquitoes, the gut microbiome of adult tsetse is dominated by *Sodalis* and *Wigglesworthia* [27], which may be reflective of the fact that tsetse feeds exclusively on sterile vertebrate blood. In an effort to understand the immunological relationship between tsetse and its microbiome, our laboratory has developed fly lines that contain altered symbiont populations. These dysbiotic fly lines, designated *Gmm^Wgm^* ^−^ and *Gmm* ^Apo^, either lack only obligate *Wigglesworthia*, or all of their symbiotic microbes, respectively, throughout their entire lifecycle. Trypanosome infection outcome experiments revealed that *Gmm^Wgm^* ^−^ individuals are significantly more susceptible to infection with trypanosomes than are their wild-type counterparts [17], [28]. This susceptible phenotype was subsequently determined to result from the fact that *Wigglesworthia*-free adults posses less trypanocidal PGRP-LB than do their parasite refractory wild-type (*Gmm* ^WT^) counterparts [17], [18]. Later studies revealed that both *Gmm^Wgm^* ^−^ and *Gmm* ^Apo^ individuals exhibit a highly irregular expression pattern of humoral and epithelial immunity-related genes and are unusually susceptible to hemocoelic infection with normally non-pathogenic *E. coli* K12. Furthermore, *Gmm^Wgm^* ^−^ flies contained a markedly deplete population of cellular immunity-associated sessile and circulating phagocytic hemocytes, while this cell type was entirely absent from aposymbiotic counterparts [29], [30]. To date no information exists regarding how *Gmm* ^Apo^ flies respond immunologically following challenge with pathogenic trypanosomes, or how this immune response subsequently influences infection outcomes.

To further our understanding of the molecular mechanisms that underlie tsetse's parasite refractory phenotype, we investigated the relationship between fly age and symbiont status as they relate to host immunity and trypanosome infection outcome. We analyzed immunity-related gene expression in *Gmm* ^WT^ teneral and mature adults, and mature *Gmm* ^Apo^ adults, following challenge with trypanosomes, and then correlated these data with the infection outcomes we observed in these distinct fly lines. Information obtained from our gene expression analysis also led to the discovery of what may be a novel mechanism that passively modulates tsetse's ability to detect, and thus subsequently respond to, immunogenic parasites. Our results provide further insights into how tsetse's endogenous symbionts regulate their host's immune response.

## Results

### Tsetse's resistance to trypanosome infection depends on fly age and microbiome status

Previous studies suggest that host age and microbiome status modulate the ability of insect disease vectors to transmit mammalian pathogens [7], [17], [20], [21]. In this study we set out to evaluate how these physiological parameters impact tsetse's ability to transmit pathogenic African trypanosomes. We began by investigating the relationship between tsetse age at the time of trypanosome challenge and subsequent infection outcomes. Previous studies have demonstrated that adult tsetse newly eclosed from their pupal case (which is known as the teneral state) are highly susceptible to infection with trypanosomes [6], [7]. We confirmed that tsetse from our laboratory colony also exhibited the ‘teneral phenomenon’ by challenging adult flies with a parasite-infective blood meal one day post-eclosion from their pupal case. We found that 54% of teneral *Gmm* ^WT^ adults harbored midgut trypanosome infections when their 1^st^ blood meal contained infective parasites (Table 1). For comparative purposes, we found that only about 3% of mature (challenged 8 days post-eclosion from their pupal case) *Gmm* ^WT^ individuals became infected with trypanosomes following the same challenge. These findings demonstrate that teneral flies from our colony are highly susceptible to infection with trypanosomes.

**Table 1:** Trypanosome infection outcomes in tsetse of differing age and symbiont status.

Treatment group	Symbiont status^a^	Infected/total # of flies^b^	Infection prevalence (%)	*p* value^c^
*Gmm* ^WT^ (teneral)^d^	*Wigglesworthia*, *Sodalis*	27/50, 17/31	54.3	<0.001
*Gmm* ^WT^ (mature)^e^	*Wigglesworthia*, *Sodalis*	3/60, 0/25, 1/23, 0/11	3.4	
*Gmm* ^Apo^ (mature)^f^	ND^g^	16/34, 5/12, 9/15, 9/13	52.7	<0.001

a Symbiotic bacteria present in tsetse's gut at the time of trypanosome challenge.

b Experiments were repeated at least twice.

c *p* values were obtained by comparing infection prevalence of each indicated group to the infection prevalence of mature *Gmm* ^WT^ flies.

d 1^st^ adult blood meal contained infectious trypanosomes.

e 4^th^ adult blood meal contained infectious trypanosomes.

f *Gmm* ^Apo^ flies underwent intrauterine larval development in the absence of all symbiotic bacteria.

g ND, no symbionts detectable by PCR.

We next investigated whether tsetse's microbiome influences fly susceptibility to trypanosomes. To do so we made use of a tsetse line, designated *Gmm* ^Apo^ (Apo, aposymbiotic), that is devoid of all of its endogenous symbiotic microbes (Figure S1A) [28], [31]. Mature *Gmm* ^Apo^ individuals were challenged with *T. b. rhodesiense* BSF trypanosomes to determine whether infection outcome correlated with the presence and composition of tsetse's microbiome. Following *per os* challenge with trypanosomes in their 4^th^ blood meal, 58% of mature *Gmm* ^Apo^ adults had established trypanosomes infections in their gut (Table 1). Conversely, as indicated above, when age-matched *Gmm* ^WT^ individuals were challenged with parasites in their 4^th^ blood meal, only 3% of flies became infected. Our discovery that mature *Gmm* ^WT^ adults are highly refractory to infection with trypanosomes, while their age-matched aposymbiotic counterparts (*Gmm* ^Apo^) are highly susceptible, strongly implies that tsetse's microbiome modulates their host's ability to mount an effective immune response following challenge with parasites. More so, the fact that both *Wigglesworthia*-free (*Gmm^Wgm^* ^−^; these flies still house commensal *Sodalis* and parasitic *Wolbachia*) [28] and *Gmm* ^Apo^ flies exhibit a similarly high susceptibility to infection with trypanosomes indicates that obligate *Wigglesworthia*, as opposed to *Sodalis* or *Wolbachia*, is the primary modulator of tsetse's immune response following challenge with pathogenic trypanosomes.

### Tsetse's adult gut microbiome does not modulate trypanosome infection outcomes

In this study we found that mature adult *Gmm^Wgm^* ^−^ and *Gmm* ^Apo^ flies are highly susceptible to infection with trypanosomes, thus indicating that tsetse's symbionts contribute to their host's immune response against challenge with this parasite. To investigate whether tsetse's gut microbiome directly or indirectly modulates its host's immune response following challenge with pathogenic trypanosomes, we fed newly emerged *Gmm* ^WT^ adults 3 blood meals supplemented with either ampicillin, to eliminate *Sodalis* from their gut, or tetracycline, which clears all endogenous microbes. Thus, these flies, which were designated *Gmm* ^WT/*Sgm−*^ and *Gmm* ^WT/Apo^, respectively, underwent intrauterine larval development in the presence of their endogenous microbiome, but existed in a dysbiotic state as adults (Figure S1B). Following the above-mentioned course of antibiotics *Gmm* ^WT/*Sgm−*^ and *Gmm* ^WT/Apo^ individuals were challenged with BSF trypanosomes. Fourteen days post-challenge we found that, similar to their mature *Gmm* ^WT^ counterparts, only about 5% of *Gmm* ^WT/*Sgm−*^ flies, and 7% of *Gmm* ^WT/Apo^ flies, were infected with trypanosomes (Table 2). Our finding that mature *Gmm* ^WT^, *Gmm* ^WT/*Sgm−*^ and *Gmm* ^WT/Apo^ flies are similarly resistant to infection with trypanosomes suggests that microbes present in the adult gut do not directly produce effector molecules, or modulate host production of effector molecules, that directly kill trypanosomes. Instead, these data suggest that the presence of the symbionts during larval maturation primes tsetse's immune system so that it develops and functions properly during adulthood.

**Table 2:** Trypanosome infection outcomes in tsetse of differing symbiont status.

Treatment group	Symbiont status^a^	Infected/total # of flies^b^	Infection prevalence (%)	*p* value^c^
*Gmm* ^WT/*Sgm*−^ (mature)^d^	*Wigglesworthia*	4/50, 0/25	5.3	0.50
*Gmm* ^WT/Apo^ (mature)^e^	ND^f^	10/118, 0/30	6.7	0.22

a Symbiotic bacteria present in tsetse's gut at the time of trypanosome challenge.

b Experiments were performed in duplicate.

c *p* values were obtained by comparing infection prevalence of each indicated group to the infection prevalence of mature *Gmm* ^WT^ flies (from Table 1).

d *Gmm* ^WT/*Sgm−*^ flies underwent intrauterine larval development in the presence of all symbiotic bacteria. Following pupal eclosion, adults received 3 blood meals supplemented with ampicillin followed by a 4^th^ containing infectious trypanosomes.

e *Gmm* ^WT/Apo^ flies underwent intrauterine larval development in the presence of all symbiotic bacteria. Following pupal eclosion, adults received 3 blood meals supplemented with tetracycline followed by a 4^th^ containing infectious trypanosomes.

f ND, no symbionts detectable by RT-PCR.

### Tsetse's immune response following challenge with trypanosomes depends on fly age and microbiome status

We determined that teneral tsetse flies are more susceptible to infection with pathogenic trypanosomes than are their mature counterparts. Additionally, we found that tsetse must harbor obligate *Wigglesworthia* during intrauterine larval development in order to overcome challenge with infectious parasites during adulthood. In an effort to better understand the association between these distinct phenotypes and the differential infection outcomes observed, we monitored the expression of immunity-related genes at two physiologically relevant time points in teneral and mature *Gmm* ^WT^ adults, and mature *Gmm* ^Apo^ adults, that were either unchallenged or challenged with trypanosomes. We chose 24 hours post-challenge (hpc) as the 1^st^ time point to determine tsetse's initial response to the presence of trypanosomes in its gut. The 2^nd^ time point, 3 days post-challenge (dpc), was chosen because a bottlenecking event at this juncture dramatically reduces trypanosome viability in tsetse's gut [32], [33]. Our prior studies demonstrated that the Imd pathway is involved in tsetse's defense against challenge with pathogenic trypanosomes [9], [10], [17]. Thus, to investigate if trypanosome challenge activated the Imd pathway in teneral and mature *Gmm* ^WT^ adults, and mature *Gmm* ^Apo^ adults, we monitored expression patterns of the associated antimicrobial peptide (AMP) effector *attacin*, as well as two negative regulators, *peptidoglycan recognition protein* (*PGRP-LB*) and *caudal*, following parasite challenge. We also evaluated the role of Jak/stat signaling by monitoring the expression of *domeless*, which is the receptor for this pathway. Finally, as indicators of cellular and epithelial immune responses, we monitored the expression patterns of two thioester-containing proteins genes (*tep2* and *tep4*), and dual oxidase (*DUOX*) and inducible nitric oxide synthase (*iNOS*), respectively. In insects TEPs presumably function as pathogen-specific opsonins that bind to foreign microbes and promote their phagocytosis or encapsulation [34], while DUOX and iNOS serve as signaling molecules that are involved in the production of reactive oxygen species and activation of humoral immune responses [35]–[37].

**Figure 1:**
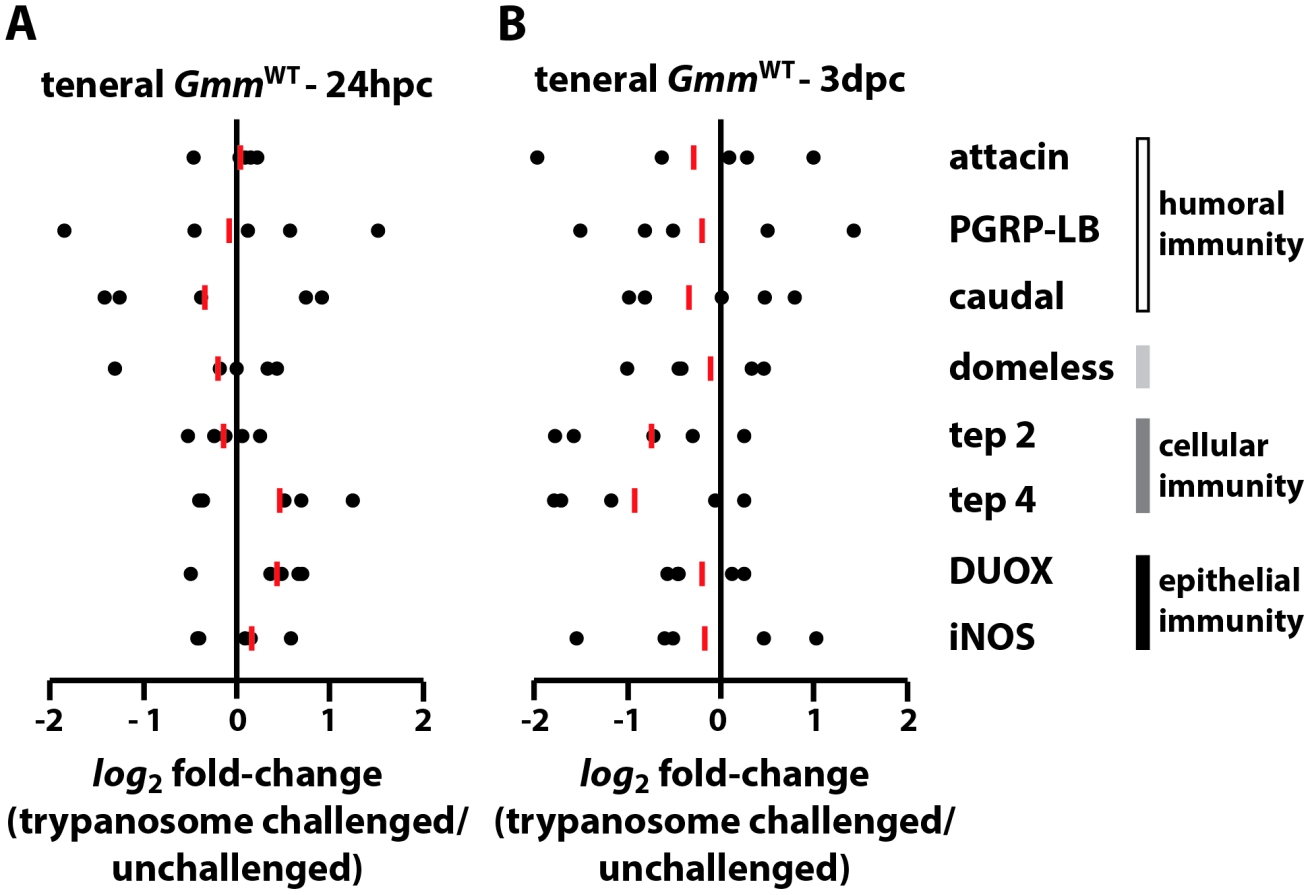
Immunity-related gene expression in teneral *Gmm* ^WT^ flies following *per os* challenge with infectious trypanosomes. *Log_2_* fold-change in the expression of immunity-related genes in teneral *Gmm* ^WT^ individuals 24 hpc (A) and 3 dpc (B) with *T. b. rhodesiense* parasites. Gene expression in challenged and unchallenged teneral *Gmm* ^WT^ individuals is normalized relative to constitutively-expressed tsetse *β-tubulin*. All l*og_2_* fold-change values are represented as a fraction of average normalized gene expression levels in trypanosome-challenged vs. unchallenged flies. Samples sizes are represented by individual dots, and the red bars indicate the median l*og_2_* fold-change for each gene assayed. All quantitative measurements were performed in duplicate. No significant difference in the expression of immunity-related genes was observed between challenged and unchallenged teneral *Gmm* ^WT^ individuals at either of the monitored time points (Student's t-test).

Expression patterns of the immunity-related genes identified above indicated that teneral *Gmm* ^WT^ adults present a highly attenuated immune response following exposure to trypanosomes at both 24 hpc (Figure 1A) and 3 dpc (Figure 1B). We argue that the immune system of teneral flies is relatively under-developed, and thus is capable of presenting only a weak response following challenge with trypanosomes. Interestingly, despite their weak immune response, ∼50% of teneral *Gmm* ^WT^ adults are able to clear trypanosomes before they establish an infection in their host's gut. This finding suggests that the effectors we examined may be functional even at low concentrations and thus inhibited the establishment of infections in 50% of challenged flies. Furthermore, our data show that the expression of *attacin*, *pgrp-lb* and *iNOS*, all of which exhibit trypanocidal activity [38], [16], [18], varies 4-fold among individual flies within the population we tested. Hence, individuals that express more of these immune molecules may be able to successfully clear their parasite infections while those with reduced levels can not.

**Figure 2:**
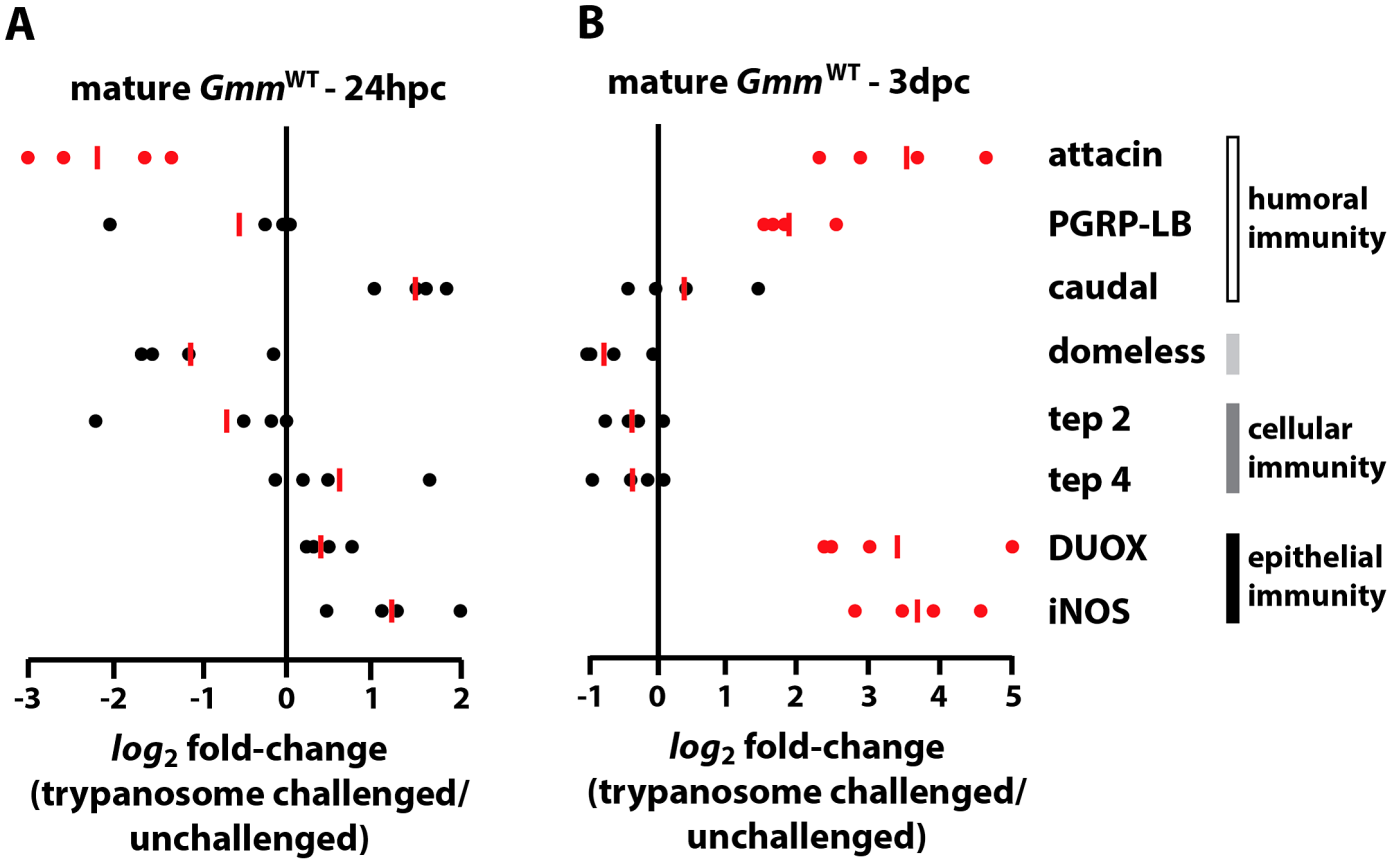
Immunity-related gene expression in mature *Gmm* ^WT^ flies following *per os* challenge with infectious trypanosomes. *Log_2_* fold-change in the expression of immunity-related genes in mature *Gmm* ^WT^ individuals 24 hpc (A) and 3 dpc (B) with *T. b. rhodesiense* parasites. Gene expression in challenged and unchallenged mature *Gmm* ^WT^ individuals is normalized relative to constitutively-expressed tsetse *β-tubulin*. All l*og_2_* fold-change values are represented as a fraction of average normalized gene expression levels in trypanosome-challenged vs. unchallenged flies. Samples sizes are represented by individual dots, and the red bars indicate the median l*og_2_* fold-change for each gene assayed. All quantitative measurements were performed in duplicate. Genes that presented a significant change in expression in parasite challenged versus unchallenged mature *Gmm* ^WT^ flies are represented by red dots (*p*≤0.05; Student's t-test).

We next compared expression patterns of the same immunity-related genes in mature adult trypanosome-resistant *Gmm* ^WT^ and trypanosome-susceptible *Gmm* ^Apo^ flies. We found that at 24 hpc *Gmm* ^WT^ adults presented a latent immune response similar to that of their younger counterparts (Figure 2A). However, by 3 dpc, the time at which parasite infections are typically beginning to clear [4], genes that encode the AMPs Attacin and PGRP-LB, as well as those that encode the epithelial immunity-related molecules DUOX and iNOS, were expressed at significantly higher levels in mature trypanosome challenged adults compared to unchallenged *Gmm* ^WT^ adults (Figure 2B). These findings suggest that multiple immune pathways contribute to the parasite resistant phenotype presented by mature *Gmm* ^WT^ adults.

**Figure 3:**
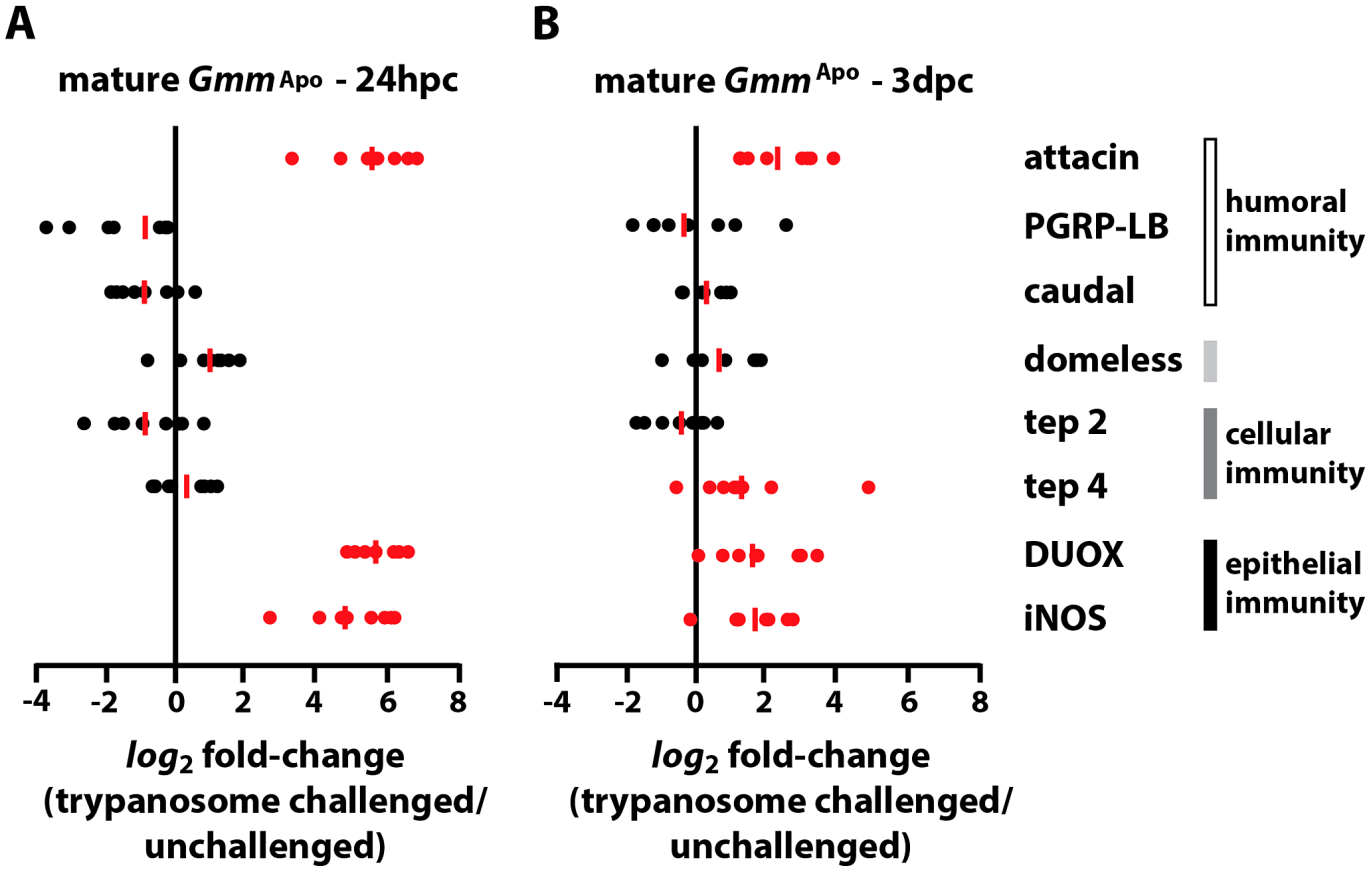
Immunity-related gene expression in mature *Gmm* ^Apo^ flies following *per os* challenge with infectious trypanosomes. *Log_2_* fold-change in the expression of immunity-related genes in mature *Gmm* ^Apo^ individuals 24 hpc (A) and 3 dpc (B) with *T. b. rhodesiense* parasites. Gene expression in challenged and unchallenged mature *Gmm* ^Apo^ individuals is normalized relative to constitutively-expressed tsetse *β-tubulin*. All l*og_2_* fold-change values are represented as a fraction of average normalized gene expression levels in trypanosome-challenged vs. unchallenged flies. Samples sizes are represented by individual dots, and the red bars indicate the median l*og_2_* fold-change for each gene assayed. All quantitative measurements were performed in duplicate. Genes that presented a significant change in expression in parasite challenged versus unchallenged mature *Gmm* ^Apo^ flies are represented by red dots (*p*≤0.05; Student's t-test).

Interestingly, *Gmm* ^Apo^ flies responded differently to challenge with trypanosomes than did their WT counterparts. In this case expression levels of *duox* and *inos*, as well as the AMP *attacin*, were significantly higher at 24 hpc in challenged compared to unchallenged individuals (Figure 3A). By 3 dpc these same genes (as well as *tep 4*) were still significantly up-regulated in parasite challenged versus unchallenged individuals. However, their median expression levels were in relative decline, suggesting that the immune response of mature *Gmm* ^Apo^ adults was in remission at this time point (Figure 3B). We hypothesize that the delayed immune activation exhibited by mature *Gmm* ^WT^ adults following exposure to trypanosomes may reflect the fact that their gut epithelium is unable to detect immunogenic parasites until at least 24 hpc. In contrast, the relatively potent immune response presented by mature *Gmm* ^Apo^ adults early in the infection process (24 hpc) suggests that the gut of these flies is able to detect the presence of parasites, and immune eliciting parasite-derived molecules, more promptly than that of their wild-type counterparts. Based on these findings we postulated that tsetse's symbionts regulate temporal aspects of the fly's ability to recognize the presence of pathogenic trypanosomes in their midgut.

### *Gmm* ^Apo^ adults present a compromised peritrophic matrix, which may enhance their susceptibility to trypanosome infections

The midgut epithelia of most insects are separated from the gut lumen by a chitinous, sheath-like structure called the peritrophic matrix (PM). Presumed functions of the insect PM include regulation of digestive processes via passive control of digestive enzyme movement into the gut lumen, protection of midgut epithelial cells from environmental toxins and mechanical damage caused by ingested food particles, and prevention or reduction in the severity of pathogen infections [39], [40]. Unlike most insects, ‘higher’ Brachyceran flies, including tsetse and *Drosophila*, house a type II PM that is constitutively produced regardless of feeding status. In the case of tsetse, this structure is immature when teneral adults emerge from their puparium. However, within 96 hrs of emergence, adult tsetse present a fully formed PM [41].

**Figure 4:**
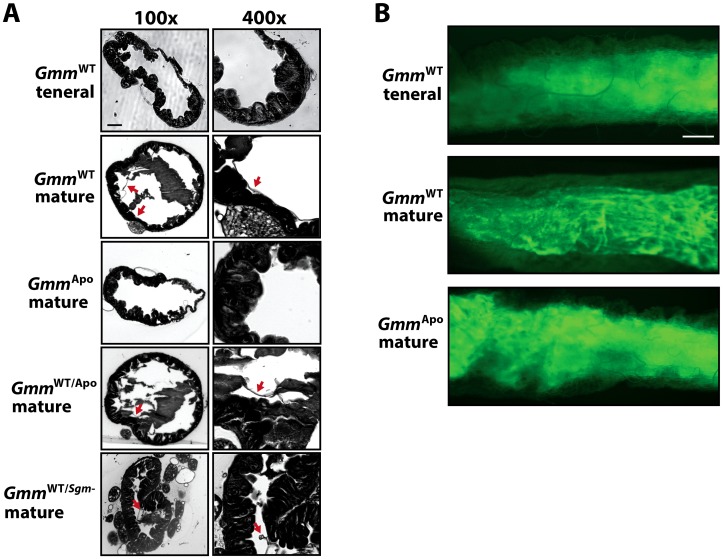
Tsetse symbiont status correlates with structural integrity of the fly's peritrophic matrix. (A) Midguts from 10 day old flies (3 days after consuming their last blood meal; *n* = 3) of each treatment group were microscopically dissected, fixed, sectioned and stained. Prepared sections were observed in an effort to compare PM structural integrity between tsetse treatment (*Gmm* ^Apo^, *Gmm* ^WT/Apo^ and *Gmm* ^WT/*Sgm*−^) and control (*Gmm* ^WT^) groups. Tsetse flies that underwent intrauterine larval developed in the presence of their endogenous microbiome (*Gmm* ^WT^, *Gmm* ^WT/Apo^ and *Gmm* ^WT/*Sgm*−^) appear to have a structurally robust PM, while those that matured in the absence of their symbionts (*Gmm* ^Apo^) do not. Red arrows identify the PM in gut sections where the structure was visible. 100× scale bars = 100 µm and 400× scale bars = 25 µm. (B) Dextran feeding assay of teneral *Gmm* ^WT^ adults, and mature *Gmm* ^WT^ and *Gmm* ^Apo^ adults (*n* = 10 per group). Flies were administered modified blood meals (see Materials and Methods, sub-section ‘Dextran feeding assay’, for details) supplemented with 500 kDa FITC-labeled dextran molecules. Six hours post-inoculation, midguts were dissected and examined under a fluorescence-emitting dissecting microscope. Scale bar (which is the same for all 3 panels) = 500 µm.

The PM from mature WT tsetse can be removed by gently grasping the structure with fine forceps and teasing it out of microscopically dissected midguts. Interestingly, we have found that when this procedure is attempted with age-matched *Gmm* ^Apo^ adults, the PM is difficult to grasp and readily breaks apart. This finding suggests that the PM of *Gmm* ^Apo^ flies may be structurally modified. Our results from experiments described above indicate that teneral *Gmm* ^WT^ adults, which lack a fully formed PM [41], and mature *Gmm* ^Apo^ adults, are similarly susceptible to infection with trypanosomes (Table 1). Thus, we hypothesized that tsetse's microbiome may modulate PM formation, which in turn affects trypanosome infection outcomes in this fly. To address this question we histologically analyzed gut tissues from teneral *Gmm* ^WT^, and mature *Gmm* ^WT^, *Gmm* ^Apo^, *Gmm* ^WT/*Sgm*−^ and *Gmm* ^WT/Apo^ adults. We observed that mature *Gmm* ^WT^, *Gmm* ^WT/*Sgm*−^ and *Gmm* ^WT/Apo^ adults have an intact PM, while this structure in age-matched *Gmm* ^Apo^ (and teneral *Gmm* ^WT^) adults is severely compromised or entirely absent (Figure 4A). To further validate that tsetse's microbiome regulates the formation of its host PM, we fed teneral *Gmm* ^WT^ adults, and mature *Gmm* ^WT^ and *Gmm* ^Apo^ adults, a modified blood meal supplemented with FITC-labeled dextran molecules (500 kDa). This procedure allowed us to visualize structural integrity of the PM by monitoring the movement of dextran through midguts of treated individuals. Six hours post-feeding, we observed that dextran molecules were contained within the PM of mature *Gmm* ^WT^ individuals. In contrast, an intact PM was absent from midguts of teneral *Gmm* ^WT^ and mature *Gmm* ^Apo^ adults, and a diffuse pattern of dextran molecules was observed in contact with surrounding intestinal epithelial tissues (Fig. 4B). Taken together, these finding indicate that tsetse's larval microbiome plays a role in the development of the adult PM. More so, young *Gmm* ^WT^ and mature *Gmm* ^Apo^ adults may be unusually susceptible to trypanosome infection because they lack a fully developed PM.

## Discussion

**Figure 5:**
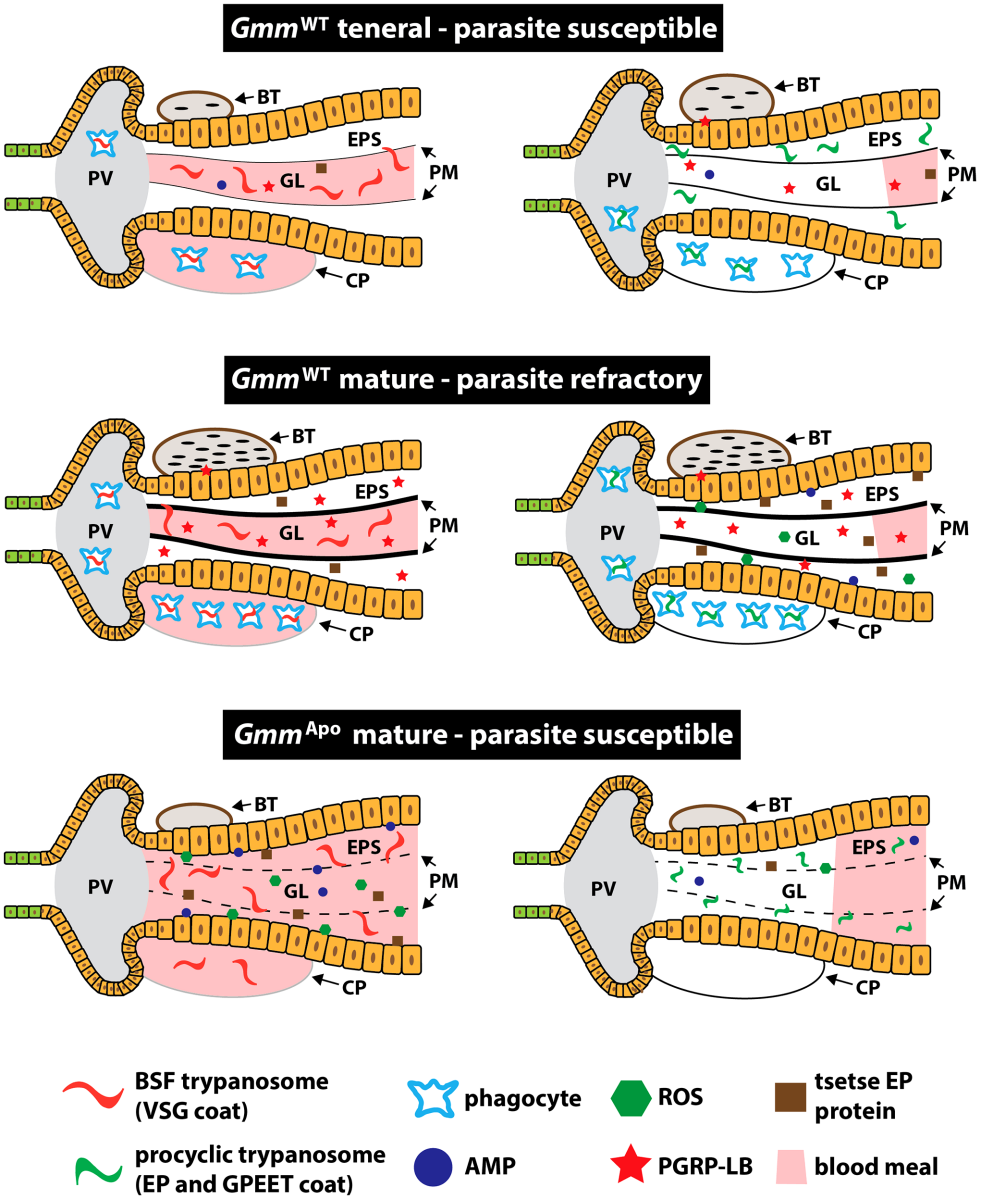
Age and symbiont status modulate trypanosome infection outcomes in the tsetse fly. Approximately 50% of teneral WT tsetse flies become infected when challenged with trypanosomes. Flies at this stage of development exhibit an immature PM, and present a weak and innocuous innate immune response following parasite challenge. Some teneral tsetse flies are refractory to parasite infections, likely because they acquire more maternally-transmitted PGRP-LB than their susceptible counterparts. Mature *Gmm* ^WT^ flies present a vigorous immune response following challenge with trypanosomes and are thus highly resistant to parasite infection. In contrast, age-matched *Gmm* ^Apo^ flies, which undergo their entire lifecycle (including intrauterine larval development) in the absence of endogenous microbes, are relatively susceptible to trypanosome infection. Although mature *Gmm* ^Apo^ flies also up-regulate the expression of several immunity-related genes following trypanosome challenge, notably absent from this list is trypanolytic *pgrp-lb*. Interestingly, the timing of this response also occurs earlier in the infection process in *Gmm* ^Apo^ compared to *Gmm* ^WT^ individuals. We propose that this premature immune response results from the fact that aposymbiotic tsetse house a structurally compromised PM that allows these flies to detect parasites immediately upon entry into the fly's midgut. Our model suggests that symbiotic microbes present in larval tsetse modulate the ability of subsequent adults to produce an intact PM. In turn, this structure regulates trypanosome infection outcomes by controlling the timing of tsetse's immune response following parasite challenge. Other invertebrates, including *D. melanogaster* and *Hirudo verbana* (the medicinal leech), house phagocytic cells in their alimentary canal that engulf pathogenic organisms [55], [56]. We speculate that WT tsetse may house similar cells in it's digestive tract that assist in the fly's immune response against trypanosome challenge. PV, proventriculus; BT, bacteriome; GL, gut lumen; EPS, ectoperitrophic space; CP, crop; PM; peritrophic matrix; AMP, antimicrobial peptide; ROS, reactive oxygen species; PGRP-LB, peptidoglycan recognition protein LB.

In the present study we provide data that further our understanding of the factors that modulate tsetse's immune response following challenge with pathogenic African trypanosomes. Based on our collective experimental evidence, we have developed a model that suggests tsetse's symbionts indirectly modulate their host's ability to detect and immunologically respond to the presence of parasites in their gut (Figure 5). We found that trypanosome-susceptible teneral *Gmm* ^WT^ flies exhibit attenuated expression of immunity-related genes following exposure to trypanosomes. In contrast, mature *Gmm* ^WT^ flies exhibit a robust immune response, regardless of adult symbiont status, and are highly resistant to parasites. Additionally, we show that mature *Gmm* ^Apo^ flies, which are also susceptible to infection with parasites, exhibit robust expression of effector genes earlier in the infection process than do their refractory, age-matched *Gmm* ^WT^ counterparts. We speculate that this untimely immune response, which appears inefficient to kill trypanosomes, may occur because mature *Gmm* ^Apo^ flies present a structurally compromised PM that permits rapid detection of parasite antigens following their entry into tsetse's gut. This novel finding demonstrates that a strong correlation exists between tsetse's larval microbiome and the integrity of the emerging adult PM. Additionally, our results indicate that this structure regulates the timing of tsetse immune induction following parasite challenge. Taken together these findings are indicative of the complex interplay that exists between tsetse's endogenous microbiome and active and passive innate immune mechanisms that influence trypanosome infection outcomes.

In this study we determine that tsetse flies from our laboratory colony exhibit the ‘teneral phenomenon’ in that approximately 50% of individuals harbor midgut infections when their first adult blood meal contains infective trypanosomes. This relatively high infection prevalence we observed in teneral *Gmm* ^WT^ adults could result from several factors. First, this population is represented by individuals that express significantly different levels of trypanolytic effectors. Those flies that produce less of these molecules may present the parasite-susceptible phenotype we observed in half of teneral individuals. The second factor may involve tsetse's PM, which is constitutively-secreted by the fly's proventriculus organ [39]. Adult flies emerge from their pupal case without a recognizable PM. At this juncture, regardless of feeding status, the matrix begins developing so that by 3–4 days post-eclosion it lines the fly's entire midgut [41]. A recent study demonstrated that trypanosome infection prevalence in tsetse's midgut was inversely related to the length of the fly's PM, as midgut infection rates were found to decrease as the time between pupal eclosion and trypanosome exposure increased [7]. The teneral flies we used for our infection study were collected over a 48 hr time frame post-emergence. Because these flies were not perfectly age-matched, the structural integrity of their PMs varied at the time of parasite challenge. Assuming that PM integrity interferes with trypanosome development in tsetse's gut, the population of trypanosome-infected teneral adults we observed may represent more recently emerged flies. Another contributory factor that may induce a parasite-susceptible phenotype in teneral adults involves maternally-derived trypanolytic immune effectors. One such molecule, PGRP-LB, is transferred to larval tsetse via female milk gland secretions. The quantity of PGRP-LB present in a teneral adult's gut positively correlates with the density of *Wigglesworthia* present in the milk gland tissue of their mother [18]. Thus, we propose that trypanosome-susceptible teneral adults inherit less PGRP-LB from their mothers than do their refractory counterparts.

In contrast to teneral *Gmm* ^WT^ adults, which exhibit a relatively ineffective immune response, mature adult tsetse employ a potent and multi-faceted active immune response following challenge with trypanosomes that likely accounts for their refractory phenotype. Immune gene expression data presented in this study indicates that *attacin*, *pgrp-lb*, *duox* and *inos* are significantly up-regulated in mature *Gmm* ^WT^ adults following trypanosome challenge. A major component of this response involves induction of immunodeficiency (Imd) pathway-associated AMPs (including Attacin). The importance of this pathway in trypanosome infection outcomes was demonstrated when AMP expression was stimulated via thoracic micro-injection with *E. coli* prior to *per os* inoculation with parasites. Tsetse that received this treatment were significantly more refractory to infection than were sham-injected controls [9]. More so, reverse genetic suppression of tsetse *pgrp-lc* and *relish*, which are components of Imd pathway, impeded induction of *attacin* and *cecropin* expression. This procedure led to an immuno-compromised phenotype characterized by a high prevalence of midgut trypanosome infections [10], [17]. Tsetse's gut also presents an epithelial immune response that appears to alter trypanosome infection outcomes in this fly. Tsetse's alimentary canal contains a distinct organ, called the proventriculus, which serves as a junction between the fly's foregut and midgut. This organ is presumably immune-responsive in that it produces cytotoxic reactive oxygen species (ROS), including nitric oxide and hydrogen peroxide, as well as Attacin and Defensin, upon microbial challenge [15]. ROS can exhibit direct anti-parasite activity, serve as signaling molecules that activate other immune pathways, and induce apoptotic cell death [37], [42]–[44]. Interestingly, trypanosome cell death can be dramatically reduced in tsetse when flies are fed a diet supplemented with a range of antioxidants [17]. This finding implicates tsetse-produced ROS as a component of the fly's trypanocidal immune response.

We found that mature *Gmm* ^Apo^ flies are significantly more susceptible to infection with trypanosomes than are age-matched WT individuals. Of note is our observation that parasitized *Gmm* ^Apo^ adults express significantly less *pgrp-lb* than do age-matched refractory *Gmm* ^WT^ individuals. This finding corroborates those from a previous study, which demonstrated that *Wigglesworthia*-free tsetse express unusually low levels of this molecule and are highly susceptible to infection with this parasite [17]. Our data also indicate that *Gmm* ^Apo^ flies, like their refractory wild-type counterparts, up-regulate the expression of immunity-related genes (*attacin*, *duox*, *inos* and *tep4*) following trypanosome challenge. However, the timing of this response occurs earlier in the infection process in *Gmm* ^Apo^ compared to *Gmm* ^WT^ individuals. Trypanocidal effector molecules produced early in the infection process would become highly diluted in the large, potentially pH-unfavorable blood meal. Furthermore, following completion of a blood meal, tsetse rapidly excretes abundant fluid volumes via diuresis [45], a process that would likely substantially decrease the quantity of soluble effector molecules present in the resulting trypanosome-containing blood bolus. Cumulatively, these conditions may account for the trypanosome-susceptible phenotype presented by mature *Gmm* ^Apo^ adults.

The comparatively early robust expression of immunity-related genes observed in mature *Gmm* ^Apo^ adults led us to hypothesize that these flies have an altered ability to immunologically detect the presence of parasite-derived antigens. As a means of addressing this theory we investigated tsetse's PM, which separates the fly's gut lumen from surrounding epithelial cells. While this structure has been proposed to serve as a barrier that physically prevents trypanosome movement through tsetse's gut [46], the genetic mechanisms that underlie PM-mediated parasite refractoriness in this fly have not been addressed. In fact, the first study to address the genetic association between the PM and host refractoriness to infection with an intestinal pathogen was recently performed using the fruit fly, *Drosophila melanogaster* [47]. In this case, *Drosophila* mutants that did not produce the protein Drosocrystallin (dcy) presented a PM that was approximately half as thick, and significantly more porous, than that found in wild-type flies. Interestingly, *dcy* mutants perished in unusually high numbers following *per os* inoculation with the entomopathogenic bacteria *Pseudomonas entomophila* and *Serratia marcescens*, as well as the pore-forming toxin Monalysin (derived from *P. entomophila*). Furthermore, mutant flies expressed significantly more of the Imd pathway-associated AMP *diptericin* than did their wild-type counterparts following oral inoculation with *P. entomophila*. These observations led to the conclusion that *Drosophila's* PM influences host infection outcomes by modulating the fly's ability to detect the presence of pathogenic organisms and the toxins they produce.

Results we present in the current study indicate that tsetse's PM serves a similar regulatory role in this fly. Specifically, we suggest that tsetse's PM influences the fly's ability to immunologically perceive and respond following challenge with parasites. When tsetse consumes an infective blood meal, stumpy BSF *T. brucei* parasites that are adapted for development in the fly midgut quickly differentiate to become procyclic forms (the majority of incompetent slender form parasites perish). This process is marked by the complete replacement of the protein coat found on the trypanosome surface [3], [48]. Thus, the early immune response presented by mature *Gmm* ^Apo^ flies may result from BSF trypanosomes, and shed surface coat molecules, having unimpeded access to immuno-reactive gut epithelia in the absence of an otherwise obstructive PM. In support of this theory, a previous study demonstrated that under normal conditions insect stage procyclic trypanosomes are not microscopically detectable in tsetse's ectoperitrophic space (EPS, the area between the PM and midgut epithelial cells) until 6 dpc [33]. Additionally, our results presented herein, and those from a previous study [9], indicate that wild-type flies that are old enough to present a fully formed PM display virtually no increase in the expression of AMPs until at least 24 hpc with BSF trypanosomes. Taken together, these results suggest that tsetse's PM does not provide a physical barrier to the passage of the parasites from the gut lumen to EPS. Instead we speculate this structure serves as a passive immune barrier that regulates tsetse's ability to immunologically detect and respond to foreign microbes in its gut. In this context, tsetse's PM likely also reduces physical contact between environmentally-acquired microbes and immune-reactive gut epithelia. This function would increase tsetse's overall fitness by preventing the induction of energetically costly immune responses that result in decreased host fecundity [49].

The association between obligate *Wigglesworthia* and tsetse immune system development is well documented. Results from this study further emphasize the steadfast nature of this association by suggesting that tsetse must house *Wigglesworthia* during larval development in order to form a fully functional PM during adulthood. Interestingly, in other insects the PM serves not only as an immune barrier, but also a biochemical one that regulates digestive and reproductive processes [39], [40], [50]. Assuming tsetse's PM exhibits similarly diverse functional roles, interfering with the structure may be exploitable as a novel form of vector control that would operate in a redundant manner to reduce this insect's capacity to transmit deadly trypanosomes.

## Materials and Methods

### Ethical consideration

This work was carried out in strict accordance with the recommendations in the Office of Laboratory Animal Welfare at the National Institutes of Health and the Yale University Institutional Animal Care and Use Committee. The experimental protocol was reviewed and approved by the Yale University Institutional Animal Care and Use Committee (Protocol 2011-07266).

### Generation of tsetse lines

Wild-type *G. morsitans morsitans* (*Gmm* ^WT^) were maintained in Yale's insectary at 24°C with 50–55% relative humidity. Throughout the manuscript, flies referred to as ‘teneral’ were unfed adults recently eclosed from their pupal case [7], while those referred to as ‘mature’ were 8 days old and had received 3 blood meals. All flies used in this study were female.

Several tsetse lines that harbored modified microbiomes were also generated for experimental use (see Table S1). The 1^st^, designated *Gmm* ^Apo^, was derived from females treated with tetracycline (20 µg per ml of blood) to clear their entire microbiome. Additionally, tetracycline-treated females also received a diet supplemented with yeast extract (1% w/v) to rescue the sterile phenotype associated with the absence of *Wigglesworthia* [30]. Thus, *Gmm* ^Apo^ offspring developed in the absence of all symbiotic bacteria (Figure S1A). Finally, 2 additional tsetse lines, designated *Gmm* ^WT/*Sgm−*^ and *Gmm* ^WT/Apo^, were generated by feeding newly eclosed *Gmm* ^WT^ adults 3 blood meals containing ampicillin (100 µg per ml of blood) or tetracycline (80 µg per ml of blood), respectively. Thus, all of these flies underwent larval development in the presence of their complete microbiome. However, as adults, *Gmm* ^WT/*Sgm−*^ individuals housed bacteriome-associated *Wigglesworthia* and intracellular *Wolbachia* (but no *Sodalis*; Figure S1B) while *Gmm* ^WT/Apo^ individuals were devoid of all symbiotic microbes (Figure S1B) [30]. All flies received defibrinated bovine blood (Hemostat Laboratories) every 48 hours through an artificial membrane feeding system [51].

### Trypanosome infections

For trypanosome infection experiments, teneral *Gmm* ^WT^ (between 24–48 hrs. old) adults received 2×10^6^ infective bloodstream form (BSF) *Trypanosoma brucei rhodesiense* per ml of blood in their 1^st^ meal. Mature *Gmm* ^WT^, *Gmm* ^Apo^, *Gmm* ^WT/*Sgm*−^ and *Gmm* ^WT/Apo^ adults received 3 trypanosome-free (but supplemented with antibiotics as indicated above) blood meals followed by a 4^th^ containing 2×10^6^ BSF *T. b. rhodesiense* per ml of blood. Fourteen days post-trypanosome challenge, all flies were dissected and their midguts microscopically examined for the presence of parasite infections. Each experiment was repeated at least twice. Replicate data were combined when no significant difference in infection prevalence was observed between individual experiments.

### Analysis of immunity-related gene expression

Immunity-related gene expression was quantified in teneral and mature *Gmm* ^WT^ adults, and *Gmm* ^Apo^ adults, 1 and 3 days post-challenge (dpc) with trypanosomes. Sample preparation and quantitative real-time PCR (qPCR) were performed as described previously [30]. Amplification primers are listed in Table S2. Quantitative measurements were performed on at least 4 biological samples (specific samples sizes are indicated in respective figure legends) in duplicate and results were normalized relative to tsetse's constitutively expressed β-tubulin gene (determined from each corresponding sample). Fold-change data are represented as a fraction of average normalized gene expression levels in trypanosome-infected flies relative to expression levels in corresponding uninfected controls. Values are represented as the mean (±SEM).

### Peritrophic matrix histological analysis

We sectioned and stained midgut tissues from mature *Gmm* ^WT^, *Gmm* ^Apo^, *Gmm* ^WT/*Sgm*−^ and *Gmm* ^WT/Apo^ adults in an effort to visually confirm PM structural integrity in these fly lines. To do so we collected guts (inclusive of the bacteriome through posterior midgut) from 10 day old individuals (*n* = 3 per tsetse treatment) 3 days after they consumed their last blood meal. Tissues were immediately fixed in Carnoy's solution (60% EtOH, 30% chloroform, 10% glacial acetic acid), embedded in agar (1.5%), dehydrated and cleared through a zylene and EtOH series, and embedded in paraffin [52]. Serial 5 µM tissue sections were cut mid-way through each midgut tissue with a rotary microtome and mounted on poly-l-lysine-coated glass slides (Richard-Allan Scientific). Prior to staining, slide-mounted samples were dewaxed through an additional zylene and EtOH series. Tissues were then stained with hematoxylin and eosin according to the manufacturer's protocol (Poly Scientific), and hard-mounted using Permount mounting solution containing toluene. Finally, samples were visualized under DIC optics using a Zeiss Axio Observer Z1 inverted microscope equipped with a Hamamatsu camera.

### Dextran feeding assay

Dextran feeding assays were performed by employing a modified version of previously described protocols [47], [53]. In brief, 500 kDa FITC-labeled dextran molecules (Sigma) were dissolved in a 2.5% sucrose solution and filtered using PD MiniTrap Sephadex G10 columns (GE Healthcare). Tsetse (*n* = 10 teneral and mature *Gmm* ^WT^, and mature *Gmm* ^Apo^) were inoculated with dextran by feeding flies a 2.5% sucrose solution containing 10% bovine blood and 10% filtered dextran molecules (1 mg/ml). Six hours post-feeding, midguts were dissected and FITC signal observed using a fluorescent dissecting microscope (Zeiss Discovery) equipped with a digital camera (Zeiss AxioCam MRc 5).

### Statistics

Statistical significance of trypanosome infection outcomes between treatment groups, and treatment and control groups, was determined using Quantitative Parasitology 3.0 [54]. Statistical analysis of qPCR data was performed by Student's t test using Microsoft Excel software.

## Supporting Information

Figure S1 **Symbiont status of tsetse flies used in this study.** (A) PCR was used to confirm that *Gmm* ^Apo^ individuals were devoid of their entire endogenous microbiome. *Gmm* ^Apo^ are offspring of antibiotic-treated moms. Thus, these flies underwent intrauterine larval development in their respective dysbiotic states. (B) RT-PCR analysis of bacterial gene expression in *Gmm* ^WT/*Sgm*−^ and *Gmm* ^WT/Apo^ flies. While individuals of these tsetse fly lines underwent intrauterine larval development in the presence of their complete endogenous microbiomes, they were treated with antibiotics during adulthood to induce dysbiosis. +C, symbiont-positive control; 1 and 2, distinct individuals from each fly line assayed to determine symbiont status.
(TIF)

Table S1 **Designation of tsetse lines used in this study, their symbiont status, and the treatment they received.**
(XLSX)

Table S2 **PCR primers used in this study.**
(XLSX)

## References

[1] Savage AF, Cerqueria GC, Regmi S, Wu Y, ElSayad NM, et al (2012) Transcript expression analysis of putative *Trypanosoma brucei* GPI-anchored surface proteins during development in the tsetse and mammalian host. PLoS Negl Trop Dis 6: e1708. 22724039 10.1371/journal.pntd.0001708 PMC3378594

[2] MacGregor P, Matthews KR (2010) New discoveries in the transmission biology of sleeping sickness parasites: applying the basics. J Mol Med 88: 865–871. 20526573 10.1007/s00109-010-0637-y PMC2921060

[3] Sharma R, Gluenz E, Peacock L, Gibson W, Gull K, et al (2009) The heart of darkness: growth and form of *Trypanosoma brucei* in the tsetse fly. Trends Parasitol 25: 517–524. 19747880 10.1016/j.pt.2009.08.001 PMC3770903

[4] Aksoy S, Gibson WC, Lehane MJ (2003) Interactions between tsetse and trypanosomes with implications for the control of trypanosomiasis. Adv Parasitol 53: 1–83. 14587696 10.1016/s0065-308x(03)53002-0

[5] Rio RV, Hu Y, Aksoy S (2004) Strategies of the home team: symbioses exploited for vector-borne disease control. Trends Microbiol 12: 325–336. 15223060 10.1016/j.tim.2004.05.001

[6] Welburn SC, Maudlin I (1992) The nature of the teneral state in *Glossina* and its role in the acquisition of trypanosome infection in tsetse. Ann Trop Med Parasitol 86: 529–536. 1288435 10.1080/00034983.1992.11812703

[7] Walshe DP, Lehane MJ, Haines LR (2011) Post eclosion age predicts the prevalence of midgut trypanosome infections in *Glossina* . PLoS One 6: e26984. 22087240 10.1371/journal.pone.0026984 PMC3210762

[8] Kubi C, Van Den Abbeele J, De Deken R, Marcotty T, Dorny P, et al (2006) The effect of starvation on the susceptibility of teneral and non-teneral tsetse flies to trypanosome infection. Med Vet Entomol 20: 388–392. 17199750 10.1111/j.1365-2915.2006.00644.x

[9] Hao Z, Kasumba I, Lehane MJ, Gibson WC, Kwon J, et al (2001) Tsetse immune responses and trypanosome transmission: implications for the development of tsetse-based strategies to reduce trypanosomiasis. Proc Natl Acad Sci USA 98: 12648–53. 11592981 10.1073/pnas.221363798 PMC60108

[10] Hu C, Aksoy S (2006) Innate immune responses regulate trypanosome parasite infection of the tsetse fly *Glossina morsitans morsitans* . Mol Microbiol 60: 1194–204. 16689795 10.1111/j.1365-2958.2006.05180.x

[11] Maudlin I, Welburn SC (1988) The role of lectins and trypanosome genotype in the maturation of midgut infections in *Glossina morsitans* . Trop Med Parasitol 39: 56–58. 3387828

[12] Welburn SC, Maudlin I, Molyneux DH (1994) Midgut lectin activity and sugar specificity in teneral and fed tsetse. Med Vet Entomol 8: 81–87. 8161852 10.1111/j.1365-2915.1994.tb00391.x

[13] Haines LR, Jackson AM, Lehane MJ, Thomas JM, Yamaguchi AY, et al (2005) Increased expression of unusual EP repeat-containing proteins in the midgut of the tsetse fly (*Glossina*) after bacterial challenge. Insect Biochem Mol Biol 35: 413–423. 15804575 10.1016/j.ibmb.2005.01.005

[14] Haines LR, Lehane SM, Pearson TW, Lehane MJ (2010) Tsetse EP protein protects the fly midgut from trypanosome challenge. PLoS Pathog 6: e1000793. 20221444 10.1371/journal.ppat.1000793 PMC2832768

[15] Hao Z, Kasumba I, Aksoy S (2003) Proventriculus (cardia) plays a crucial role in immunity in the tsetse fly. Insect Biochem Mol Biol 33: 1155–1164. 14563366 10.1016/j.ibmb.2003.07.001

[16] MacLeod ET, Maudlin I, Darby AC, Welburn SC (2007) Antioxidants promote establishment of trypanosome infections in tsetse. Parasitol 134: 827–831. 10.1017/S0031182007002247 17306056

[17] Wang J, Wu Y, Yang G, Aksoy S (2009) Interactions between mutualist *Wigglesworthia* and tsetse peptidoglycan recognition protein (PGRP-LB) influence trypanosome transmission. Proc Natl Acad Sci USA 106: 12133–12138. 19587241 10.1073/pnas.0901226106 PMC2715537

[18] Wang J, Aksoy S (2012) PGRP-LB is a maternally transmitted immune milk protein that influences symbiosis and parasitism in tsetse's offspring. Proc Natl Acad Sci USA 109: 10552–10557. 22689989 10.1073/pnas.1116431109 PMC3387098

[19] Xi Z, Ramirez JL, Dimopoulos G (2008) The *Aedes aegypti* toll pathway controls dengue virus infection outcomes. PLoS Pathogens 4: e1000098. 18604274 10.1371/journal.ppat.1000098 PMC2435278

[20] Cirimotich CM, Ramirez LJ, Dimopoulos G (2011) Native microbiota shape insect vector competence for human pathogens. Cell Host Microbe 10: 307–310. 22018231 10.1016/j.chom.2011.09.006 PMC3462649

[21] Weiss B, Aksoy S (2011) Microbiome influences on host vector competence. Trends Parasitol 27: 514–522. 21697014 10.1016/j.pt.2011.05.001 PMC3179784

[22] Cirimotich CM, Dong Y, Clayton AM, Sandiford SL, Souza-Neto SL, et al (2011) Natural microbe-mediated refractoriness to *Plasmodium* infection in *Anopheles gambiae* . Science 322: 855–858. 10.1126/science.1201618 PMC4154605 21566196

[23] Dong Y, Manfredini F, Dimopolous G (2009) Implications of the mosquito midgut microbiota in the defense against malaria parasites. PLoS Pathog 5: e1000423. 19424427 10.1371/journal.ppat.1000423 PMC2673032

[24] Rodrigues J, Brayner FA, Alves LC, Dixit R, Barillas-Mury C (2010) Hemocyte differentiation mediates innate immune memory in *Anopheles gambiae* mosquitoes. Science 329: 1353–1355. 20829487 10.1126/science.1190689 PMC3510677

[25] Aksoy S (2000) Tsetse – a haven for microorganisms. Parasitol Today 16: 114–118. 10689331 10.1016/s0169-4758(99)01606-3

[26] Attardo GM, Lohs C, Heddi A, Alam UH, Yildirim S, et al (2008) Analysis of milk gland structure and function in *Glossina morsitans*: milk protein production, symbiont populations and fecundity. J Insect Physiol 54: 1236–1242. 18647605 10.1016/j.jinsphys.2008.06.008 PMC2613686

[27] Maltz MA, Weiss BL, O'Neill M, Wu Y, Aksoy S (2012) OmpA-mediated biofilm formation is essential for the commensal bacterium *Sodalis glossinidius* to colonize the tsetse fly gut. App Environ Microbiol 78: 7760–7768. 10.1128/AEM.01858-12 PMC3485708 22941073

[28] Pais RR, Lohs C, Wu Y, Wang J, Aksoy S (2008) The obligate mutualist *Wigglesworthia glossinidia* influences reproduction, digestion and immunity processes of its host, the tsetse fly. App Environ Microbiol 74: 5965–5974. 10.1128/AEM.00741-08 PMC2565960 18689507

[29] Weiss BL, Wang J, Aksoy S (2011) Tsetse immune system maturation requires the presence of obligate symbionts in larvae. PLoS Biol 9: e1000619. 21655301 10.1371/journal.pbio.1000619 PMC3104962

[30] Weiss BL, Maltz M, Aksoy S (2012) Obligate symbionts activate immune system development in the tsetse fly. J Immunol 188: 3395–3403. 22368278 10.4049/jimmunol.1103691 PMC3311772

[31] Alam U, Medlock J, Brelsford C, Pais R, Lohs C, et al (2011) *Wolbachia* symbiont infections induce strong cytoplasmic incompatibility in the tsetse fly, *Glossina morsitans morsitans* . PLoS Pathog 7: e1002415. 22174680 10.1371/journal.ppat.1002415 PMC3234226

[32] Van den Abbeele J, Claes Y, Van Bockstaele D, Le Ray D, Coosemans M (1999) *Trypanosoma brucei* spp. development in the tsetse fly characterization of the post-mesocyclic stages in the foregut and proboscis. Parasitol 118: 469–478. 10.1017/s0031182099004217 10363280

[33] Gibson W, Bailey M (2003) The development of *Trypanosoma brucei* within the tsetse fly midgut observed using green fluorescent trypanosomes. Kinetoplastid Biol Dis 2: 1–13. 12769824 10.1186/1475-9292-2-1 PMC156611

[34] Blandin SA, Levashina EA (2007) Phagocytosis in mosquito immune responses. Immunol Rev 219: 8–16. 17850478 10.1111/j.1600-065X.2007.00553.x

[35] Ha E, Oh C, Bae YS, Lee W (2005) A direct role for dual oxidase in *Drosophila* gut immunity. Science 310: 847–850. 16272120 10.1126/science.1117311

[36] Kumar S, Molina-Cruz A, Gupta L, Rodrigues J, Barillas-Mury C (2010) A peroxidase/dual oxidase system modulates midgut epithelial immunity in *Anopheles gambiae* . Science 327: 1644–1648. 20223948 10.1126/science.1184008 PMC3510679

[37] Foley E, O'Farrell PH (2003) Nitric oxide contributes to induction of innate immune responses to gram-negative bacteria in *Drosophila* . Genes Dev 17: 115–125. 12514104 10.1101/gad.1018503 PMC195964

[38] Hu Y, Aksoy S (2005) An antimicrobial peptide with trypanocidal activity characterized from *Glossina morsitans morsitans* . Insect Biochem Mol Biol 35: 105–115. 15681221 10.1016/j.ibmb.2004.10.007

[39] Lehane MJ (1997) Peritrophic matrix structure and function. Ann Rev Entomol 42: 525–550. 15012322 10.1146/annurev.ento.42.1.525

[40] Hegedus D, Erlandson M, Gillot C, Toprak U (2009) New insights into peritrophic matrix synthesis, architecture, and function. Annu Rev Entomol 54: 285–302. 19067633 10.1146/annurev.ento.54.110807.090559

[41] Lehane MJ, Msangi AR (1991) Lectin and peritrophic membrane development in the gut of *Glossina m. morsitans* and a discussion of their role in protecting the fly against trypanosome infection. Med Vet Entomol 5: 495–501. 1773127 10.1111/j.1365-2915.1991.tb00578.x

[42] Dimopoulos G, Richman A, Muller HM, Kafatos FC (1997) Molecular immune responses of the mosquito *Anopheles gambiae* to bacteria and malaria parasites. Proc Natl Acad Sci USA 94: 11508–11513. 9326640 10.1073/pnas.94.21.11508 PMC23521

[43] Luckhart S, Vodovotz Y, Cui L, Rosenberg R (1998) The mosquito *Anopheles stephensi* limits malaria parasite development with inducible synthesis of nitric oxide. Proc Natl Acad Sci USA 95: 5700–5705. 9576947 10.1073/pnas.95.10.5700 PMC20442

[44] Hildeman DA (2004) Regulation of T-cell apoptosis by reactive oxygen species. Free Rad Biol Med 36: 1496–1504. 15182852 10.1016/j.freeradbiomed.2004.03.023

[45] Gee JD (1975) Diuresis in the tsetse fly *Glossina austeni* . J Exp Biol 63: 391–401. 1202130 10.1242/jeb.63.2.381

[46] Ellis DS, Evans DA (1977) Passage of *Trypanosoma brucei rhodesiensis* through the peritrophic membrane of *Glossina morsitans morsitans* . Nature 267: 834–835. 895841 10.1038/267834a0

[47] Kuraishi T, Binggeli O, Opota O, Buchon N, Lemaitre B (2011) Genetic evidence for a protective role of the peritrophic matrix against bacterial infection in *Drosophila melanogaster* . Proc Natl Acad Sci USA 108: 15966–15971. 21896728 10.1073/pnas.1105994108 PMC3179054

[48] Roditi I, Lehane MJ (2008) Interactions between trypanosomes and tsetse flies. Curr Opin Microbiol 11: 345–351. 18621142 10.1016/j.mib.2008.06.006

[49] Hu C, Rio RV, Medlock J, Haines LR, Nayduch D, et al (2008) Infections with immunogenic trypanosomes reduce tsetse reproductive fitness: potential impact of different parasite strains on vector population structure. PLoS Negl Trop Dis 2: e192. 18335067 10.1371/journal.pntd.0000192 PMC2265429

[50] Shao L, Devenport M, Jacobs-Lorena M (2004) The peritrophic matrix of hematophagous insects. Arch Insect Biochem Physiol 47: 119–125. 10.1002/arch.1042 11376458

[51] Moloo SK (1971) An artificial feeding technique for *Glossina* . Parasitol 68: 507–512. 10.1017/s0031182000080021 5139030

[52] Kikuchi Y, Graf J (2007) Spatial and temporal dynamics of a naturally occurring two-species microbial community inside the digestive tract of the medicinal leech. Appl Environ Microbiol 73: 1984–1991. 17277211 10.1128/AEM.01833-06 PMC1828818

[53] Edwards MJ, Jacobs-Lorena M (2000) Permeability and disruption of the peritrophic matrix and caecal membrane from *Aedes aegypti* and *Anopheles gambiae* mosquito larvae. J Insect Physiol 46: 1313–1320. 10844150 10.1016/S0022-1910(00)00053-6

[54] Rozsa L, Reiczigel A, Majoros G (2000) Quantifying parasites in samples of hosts. J Parasitol 86: 228–232. 10780537 10.1645/0022-3395(2000)086[0228:QPISOH]2.0.CO;2

[55] Zaidman-Remy A, Regan JC, Brandao AS, Jacinto A (2012) The *Drosophila* larva as a tool to study gut-associated macrophages: PI3K regulates a discreet hemocytes population at the proventriculus. Dev Comp Immunol 36: 638–647. 22085781 10.1016/j.dci.2011.10.013

[56] Silver AC, Kikuchi Y, Fadl AA, Sha J, Chopra AK, Graf J (2007) Interaction between innate immune cells and a bacterial type III secretion system in mutualistic and pathogenic associations. Proc Natl Acad Sci USA 104: 9481–9486. 17517651 10.1073/pnas.0700286104 PMC1890520

