# Supplementary figures and images for: Trypanosome Infection Establishment in the Tsetse Fly Gut Is Influenced by Microbiome-Regulated Host Immune Barriers
Source: PLoS Pathog. 2013 Apr 18;9(4):e1003318. doi: 10.1371/journal.ppat.1003318 (PMC3630092; doi:10.1371/journal.ppat.1003318)

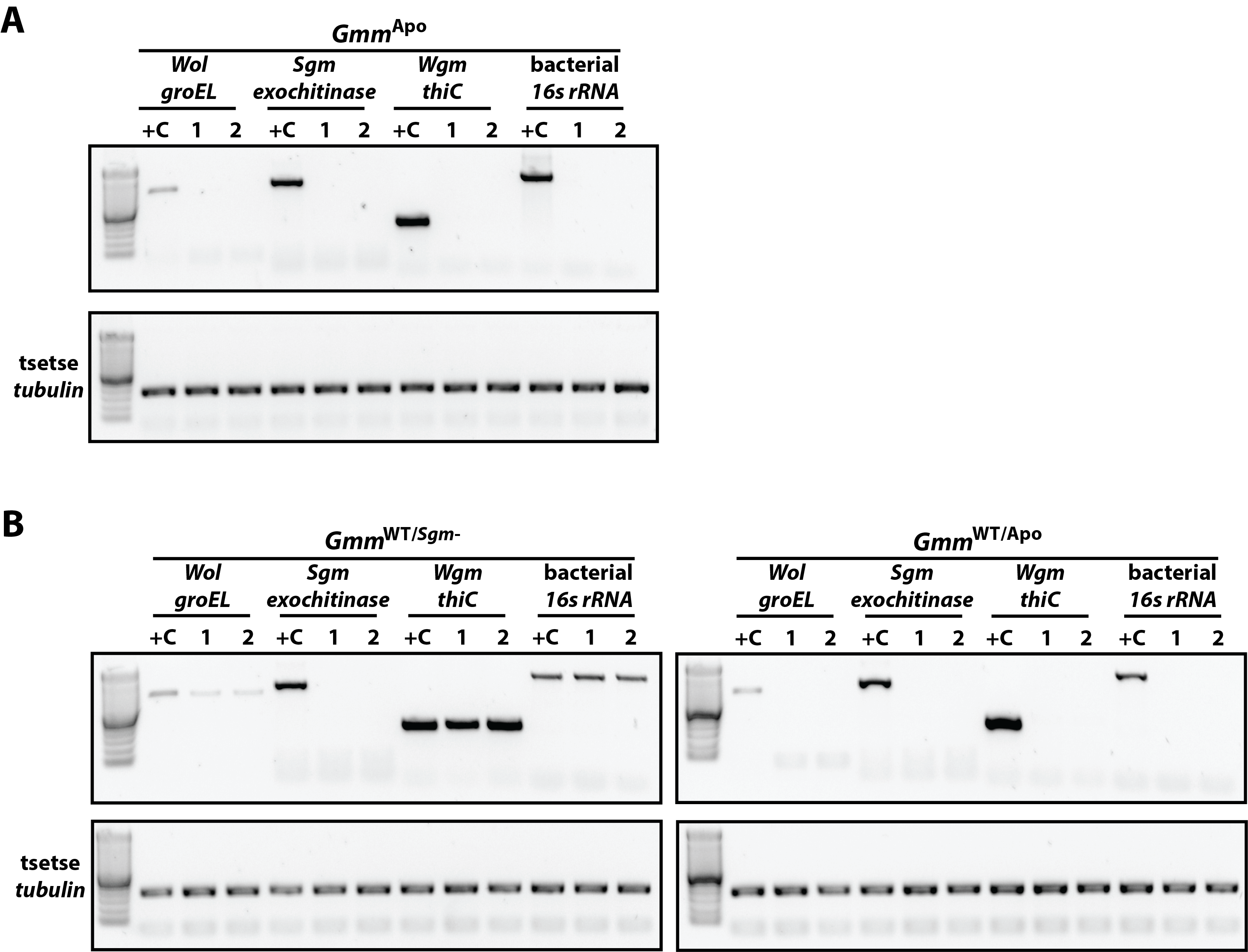

Supplement: Figure S1 — Symbiont status of tsetse flies used in this study. (A) PCR was used to confirm that Gmm Apo individuals were devoid of their entire endogenous microbiome. Gmm Apo are offspring of antibiotic-treated moms. Thus, these flies underwent intrauterine larval development in their respective dysbiotic states. (B) RT-PCR analysis of bacterial gene expression in Gmm WT/Sgm− and Gmm WT/Apo flies. While individuals of these tsetse fly lines underwent intrauterine larval development in the presence of their complete endogenous microbiomes, they were treated with antibiotics during adulthood to induce dysbiosis. +C, symbiont-positive control; 1 and 2, distinct individuals from each fly line assayed to determine symbiont status. (TIF) [file ppat.1003318.s001.tif]
